# ‘There You Enjoy Life, Here You Work’: Brazilian and Dominican Immigrants’ Views on Work and Health in the U.S.

**DOI:** 10.3390/ijerph16204025

**Published:** 2019-10-21

**Authors:** Cristina Araujo Brinkerhoff, C. Eduardo Siqueira, Rosalyn Negrón, Natalicia Tracy, Magalis Troncoso Lama, Linda Sprague Martinez

**Affiliations:** 1 School of Social Work, Boston University, Boston, MA 02215, USA; Crisb@bu.edu; 2 Department of Environment and Public Health, University of Massachusetts Boston, Boston, MA 02125, USA; carlos.siqueira@umb.edu; 3 Anthropology Department, University of Massachusetts Boston, Boston, MA 02125, USA; Rosalyn.negron@umb.edu; 4 Brazilian Worker Center, Boston, MA 02134, USA; NAT-TRACY@msn.com; 5 Dominican Development Center, Boston, MA 02130, USA; magatroncoso@msn.com

**Keywords:** Brazilian, Dominican, immigrants, work, health

## Abstract

Structural inequalities in the U.S. work environment place most immigrants in low paying, high-risk jobs. Understanding how work experiences and influence the health of different immigrant populations is essential to address disparities. This article explores how Brazilian and Dominican immigrants feel about their experiences working in the U.S. and how the relationship between work and culture might impact their health. In partnership with the Dominican Development Center and the Brazilian Worker Center, we held five cultural conversations (CCs) with Brazilians (*n* = 48) and five with Dominicans (*n* = 40). CCs are participatory, unstructured groups facilitated by representatives from or embedded in the community. Brazilian immigrants focused on physical health and the American Dream while Dominicans immigrants emphasized concerns about the influence of work on mental health. Dominicans’ longer tenure in the U.S. and differences in how Brazilians and Dominicans are racialized in the region might account for the variation in perspectives between groups. Future studies should further investigate the relationship between health and how immigrants’ work lives are shaped by culture, race and immigrant status.

## 1. Introduction

Structural inequities in the United States (U.S.) have an effect on the lives and health of immigrants in addition to U.S. born communities of color [1,2]. Labor-force segregation and immigrants’ social, political and, educational circumstances can funnel immigrants towards hazardous low-income occupations [1,3]. New immigrants often experience exposure to physical and chemical hazards as well as oppressive work conditions in which they are mistreated, underpaid or not paid at all [4,5,6,7]. In addition, a shift from a diffuse and collective culture of work to a more individualistic and segmented work environment that characterizes the experience of many immigrants is also a stressor that impacts their wellbeing [8]. Immigrants have to adapt to a new culture, new language and new environment, which are known stressors, especially for those without legal documentation who fear deportation [9]. From an ecological point of view, immigrants have compounding socio-cultural, economic and workplace disadvantages that may explain the health declines seen overtime [10]. These disadvantages are a threat to immigrants’ health and wellbeing.

On average, people employed full time in America spend an estimated 8.4 h at work per day [11]. As such, work has been identified as an important determinant of health [1,12]. Work, however, has both positive and negative implications for health. For example, the literature on aging indicates work can be associated with a sense of purpose and social engagement which translates to improved health and psychological wellbeing [13,14]. At the same time, the stress associated with work and the work environment contributes to a variety of disorders [1,15]. Work-related risks and benefits have been associated with social class and professional status [16]. New immigrants often face significant health risks associated with work; many occupy the bottom of the economic ladder and are overrepresented in jobs that pose a danger of injury or exposure to hazards, such as construction, domestic workers, restaurant workers, nail salon workers and farm work [4,5,6,17,18,19,20,21,22].

Understanding the work experiences of different immigrant populations and the association between workplace exposures and health and wellbeing is a vital step to addressing workplace conditions. Previous research on the work experiences of Latinos in the Midwest revealed that participants worked demanding and difficult jobs for survival and power, social connections and self-determination, and faced barriers spanning from legal status to English proficiency [23]. Additional research on Latina’s work experience and found high workloads, cultural tension, hazards, lack of access to healthcare and of work-family balance were threats to their wellbeing [24]. However, the Latino population is not monolithic and there is much diversity between and within populations. Dominican immigrants are often included in research as “Latino” while there are only a few studies that explore the work experiences of Brazilian immigrant workers [25,26]. Thus, the available literature on work among Latino immigrants is limited in its representation of the richness of the experiences of individual subpopulations.

We add to the literature by presenting an ecological perspective on the intersection between work, culture and health among Brazilian and Dominican immigrants living in the Greater Boston area. As part of a larger community engaged study of transnationalism, social networks, culture and health, we set out to identify factors that influence health in the context of migration for Brazilian and Dominican immigrants. Work, societal and cultural norms regarding work in the United States, working conditions and the work environment emerged as salient themes related to health and wellbeing for both Brazilian and Dominicans immigrants in our sample. This article addresses the following questions: how do Brazilians and Dominicans feel about their experiences working in the U.S. and how might cultural dimensions and work experiences interact to impact their health?

Using data from focus groups, conducted in partnership with the Brazilian Worker Center (BWC) and Dominican Development Center (DDC), which we framed as cultural conversations (CCs), with Brazilian and Dominican immigrants, we examine their perceptions of work in the U.S. and beliefs surrounding the relationship between work, health and culture. We also assessed differences and similarities between both communities’ experiences and attitudes towards work in the U.S. Although we have individual level data, we frame our understanding of immigrants’ health at structural levels emphasizing the intersection of culture, immigration, work and social class and how these forces impact immigrants’ health outcomes [27]. We discuss the narratives in the context of Brazilian and Dominican immigrants’ demographics and findings from previous studies. Next, we present a brief background on our study population and work-related health risks experienced by Brazilian and Dominican immigrants is followed by the study methodology, results and a discussion of our findings.

### 1.1. Brazilian and Dominican Immigrants in Massachusetts

Brazilians and Dominicans represent two of the largest immigrant communities in Massachusetts, this is particularly true in the Greater Boston area. In 2017, there were an estimated 95,946 Brazilians and 172,577 Dominicans living in Massachusetts [28,29]. Both communities are racially diverse, overrepresented among the population in poverty, and disproportionately impacted by chronic health conditions over time [10]. Brazilians and Dominicans are also considered highly transnational populations because of their strong economic, political, social and familial ties and investments in the U.S. and countries of origin. The transnational character of their communities is also seen in the significant immigrant engagement in country of origin politics, as with Dominicans in Boston [30], and the enthusiastic consumption of popular culture products including home country *telenovelas*, magazines, and televised soccer, as Margolis (1995) observed among Brazilian immigrants [31]. However, each populations’ history of migration to the U.S. and social integration patterns are quite different, as described next.

### 1.2. Brazilian Immigrants and Work

Brazil is a nation of continental proportions with a population size relatively comparable to that of the U.S. However, emigration is a relatively new phenomenon starting circa 1964 with political volatility and fear of violence resulting from the military coup [32]. Over time, links created by U.S. multinational endeavors paired with economic instability prompted a larger migration flows in the 1980s and mid 2000s [32,33]. Brazilian migration patterns have been referred to as a “yo-yo” due to constant comings and goings, however, as visa restrictions were applied to Brazilian nationals, over time they became more settled in the U.S. [33]. There are now almost half a million foreign-born Brazilians living in the U.S. [34]. Among those, it is estimated that more than 100,000 are undocumented [35], which is a reason for concern for these populations’ physical and mental health and wellbeing [36]. The vast majority of Brazilian immigrants were not here to benefit from the Immigration Reform and Control Act’s (IRCA) amnesty provision in 1986, which has left a large immigrant population in decades-long limbo [32,33]. The lack of legal documentation has implications for the job opportunities Brazilians can pursue in the U.S. and their access to health insurance.

Brazilian immigrants are also overrepresented in hazardous occupations, suffer exploitation in the workplace, including long work hours with few breaks and wage theft [4,5,25]. Previous researchers have found that Brazilians are largely employed in service and constructions sectors and are highly vulnerable to work pressure from employers, early and irregular shifts, as well as lack of job, health and safety training [4,5,25,26]. Research also revealed high exposure to chemical and physical hazards especially for the large population of Brazilian immigrants who perform cleaning jobs and construction work [4,5,17,25,37]. The consequences of these work environments on health outcomes are dire, with high rates of endorsement by Brazilian immigrants of musculoskeletal problems, respiratory problems, headaches, exhaustion, sleeping problems as well as psychological and mood behavioral issues such as irritability and periods of depression [4,17,25].

To our knowledge, only two studies qualitatively assessed the experiences of Brazilian immigrants in the American work environment. Messias (2001) investigated how Brazilian immigrant women who perform domestic and cleaning work perceived their experiences working in the U.S. and the relationships between work and culture in the intersection of their multiple identities. Pannikar and collegues (2015) explored the hazardous features of Brazilian immigrant women’s work and the consequences to these for women’s health and wellbeing. While the current qualitative literature provides a rich and in-depth look into the work experiences of Brazilian immigrant women, additional research inclusive of different work environments and work experiences is needed. Thus, one aim of our study is to further illuminate how Brazilian immigrants from diverse work environments understand working in the U.S., the different cultural lenses through which precarious work environments are understood, and how such experiences compare with that of other immigrants, such as Dominicans.

### 1.3. Dominican Immigrants and Work

Dominican migration to the U.S. started around 1961 with the death of the dictator Rafael Trujillo at the hands of rebel forces [38]. More than half of all Dominican immigrants arrived before 2000 [38]. Today there are more than 2 million Dominican-born immigrants living in the U.S., which means that approximately 1 in 7 Dominicans are living outside the island [39,40]. In contrast to Brazilians, more than half of foreign-born Dominican immigrants are naturalized U.S. citizens [41]. Family reunification, which can only be achieved through naturalization, is an important pathway to legalization for this population [38]. Despite the higher rates of citizenship, Dominicans are more likely to live in poverty and to have significantly lower levels of educational attainment compared to other Latino populations counterparts and the native-born population [38,41].

In Boston, about 34% of Dominicans work in service occupations, 23% work in production, transportation and material moving occupations, 19% work in technical, sales, and administrative support occupations, and about 8% work in construction or maintenance [29]. Dominican men most often work in construction and manufacturing industries, while the majority of Dominican women work in the personal care, sales, and services industries [42]. Less than 9% of Dominicans in the Boston area work in managerial positions, which is significantly lower than both U.S.-born residents and other immigrant groups [42]. This is all despite the fact that the majority of Dominicans immigrate for greater socioeconomic opportunity [42]. The economic difficulties faced by Dominicans living in the United States are likely tied to the same barriers experienced by most Latino and immigrant populations: discrimination and prejudice based on factors such as perceived unauthorized documentation status and English-language abilities. Many members of the Dominican community identifying as black or Afro-Caribbean also experience racism based on skin color, which most other segments of the Latino population do not experience [43]. As such, the Dominicans living in the U.S. are more likely to report incidents of racial discrimination as compared to other Latino/a populations [44]. Experiences of racism and prejudice are known to increase stress and other mental health symptoms in the Dominican community [44,45].

This study contributes to the literature on Dominican immigrants by presenting the communities’ perceptions of work and the burdens of work for their health. Previous research explored the experiences of Dominican immigrant women balancing work in the U.S. and homemaking in a patriarchal culture and found that Dominican women are motivated by the family separating themselves from the working class, and participating in organized workplace resistance [46]. However, there are no studies to our knowledge that qualitatively explore the relationships between work and health for Dominican immigrants. This study aims to begin to bridge this gap.

## 2. Materials and Methods

This article draws on data from a community engaged project that was a joint effort between the BWC, the DDC and three Massachusetts universities: University of Massachusetts Boston, Boston University and Tufts University. The project’s aim was to explore social networks, transnationalism, culture and health for Brazilians and Dominicans in the greater Boston area. We conducted five unstructured focus groups with Brazilians and five with Dominicans, which we called cultural conversations (CCs). This study was approved by the Boston University Charles River Campus Institutional Review Board and the Tufts University Social Science, Behavioral and Education Research Institutional Review Board.

### 2.1. Sampling and Recruitment

A non-probability purposive sampling strategy was employed to identify participants for each phase of the research. In collaboration with community partners, the BWC and the DDC, we enlisted the participation of Brazilian and Dominican immigrants who were the first or 1.5 generations. First-generation immigrants are foreign born individuals who migrate to the U.S. as adults while 1.5 generation are foreign born individuals who migrate as children or young teens. Brazilian (*n* = 48) and Dominican (*n* = 40) participants were recruited in English to Speakers of Other Languages (ESOL) classes, barbershops, community and religious events as well as word of mouth. We recruited and placed participants into groups based on criteria that reflected key socio-demographic and occupational characteristics of both communities. For instance, previous knowledge that low-income Brazilian women are overrepresented in domestic work occupations in the Greater Boston led us to outreach to domestic workers for one of the conversations. The different CCs included elders (Brazilians 60+), professionals (Brazilians and Dominicans), low-wage workers (Dominicans), domestic workers (Brazilians), barbers (Dominicans), construction workers (Brazilians), and generation 1.5 (Brazilians and Dominicans). All participants were provided informed consent forms and explanation of their rights on their preferred language (English, Portuguese or Spanish).

### 2.2. Cultural Conversations

CCs are reflective group discussions steeped in critical pedagogy [47]. Prompts by the facilitator guided participants to speak about cultural artifacts, cultural practices, culture change, and the impact of culture on health and behaviors in the context of immigration. CCs were participatory, unstructured, and facilitated by representatives from or embedded in the target community. For instance, CCs were facilitated by the BWC and DDC executive directors, a Brazilian academic and graduate student. Conversations were allowed to progress organically and lasted an average of one hour and half hours. Participants guided the dialogue often challenging and posing additional questions to one another. The ability and freedom given to participants to share the steering wheel facilitated a more equitable group dynamic. Shared group power and the recognition of individual knowledge are key components of critical pedagogy. Work and behaviors related to work emerged as salient themes across most Brazilian and Dominican groups, despite the absence of direct prompts focused on work. Most cultural conversations were conducted in Portuguese or Spanish except for the generation 1.5 groups that felt more comfortable with English or a combination of both languages.

### 2.3. Analyses

Well-trained bilingual researchers transcribed each CC group verbatim in the language in which data were collected (Brazilian Portuguese, Spanish or English) and uploaded the transcripts into NVivo ® (QSR International, Melbourne, Australia). Thematic analyses were used to explore the CC transcripts, initially using a deductive approach based on prompts from the guide and codes related to culture such as “definitions of culture”, “changes in culture” and “influence of migration on beliefs and behavior” [48]. Researchers and community partners met regularly to share, discuss and contextualize initial findings. Next, the researchers used their deep knowledge of the data, community members and research staff insights to further code the CCs through an inductive approach. We then focused our coding related to work experiences, working conditions and individual behaviors using NVivo word queries (Table 1). The first and last authors reviewed all transcripts to check for the words highlighted in the queries as well as additional passages related to work experience and health. Quotes were compiled and coded inductively for initial themes [48]. The themes were then refined for each immigrant group to show similarities and differences in work experiences.

## 3. Results

Conversations were organized according to socio-demographic characteristics relevant for each population (Table 2). Participants in the CC with Brazilians and Dominicans, professional groups were composed of healthcare and middle management professionals that work in the Greater Boston area. The 1.5 generation Brazilian conversation was composed of college students, most of whom benefited from Deferred Action for Childhood Arrival (DACA). The Dominican 1.5 generation participants were part-time community college students and two recent graduates from professional graduate programs. The participants in the conversation with Brazilian domestic workers were immigrant women who worked as housecleaners or nannies, while those in the Conversation with low-wage workers were female Dominicans who were largely child care providers, health care aids, community volunteers and teaching assistants. Participants in the Conversation with male Brazilians were construction workers and those in the Conversation with Dominicans were barbers. Dominican Elders were recruited through the Southern Jamaica Plain Health Center (SJPHC). Since the Brazilian population is relatively young, we defined Brazilian elders as those older than 60 years old. They were recruited by BWC in collaboration with a cultural center in Framingham, Massachusetts. CCs were held at the Brazilian Worker Center, the Dominican Development Center, UMass Boston, a local barber shop, a Cultural Center in Framingham, and the SJPHC.

Work was a salient theme that animated all CC groups but the Brazilian Elders (60+), who were more concerned about immigrant experiences with adaptation (language, culture), health behaviors and healthcare. Thus, we excluded that transcript from our analyses.

### 3.1. Live to Work vs. Work to Live

Brazilians and Dominicans described dramatic shifts in work life balance, because after migration to the U.S their lives were centered around work. We found four significant themes in our analyses: Go, Go Culture, American Dream, Work Conditions, and Work and Health. Brazilians and Dominicans in different CCs had various rates of endorsement of these concepts illustrating the diversity of experiences in these communities.

### 3.2. Go, Go Culture

Brazilian immigrants created a unique word for the hectic condition that most immigrants find themselves in daily: “bisado” (pronounced: bee-zah-doh), which is a Brazilian Portuguese version of the word busy. When it comes to work, Brazilians described a routine that was not compatible with leisure or health promoting activities. A participant in the construction worker CC stated, “I just work, go home, and crash. The next day I get up and go to work again. I do not do any physical exercise.” The “go, go” aspect of American culture was present in most CCs, and the difference in the rhythm of life between the U.S. and Brazil was often mentioned. A participant in the domestic workers’ CC summarized it:

“We Brazilians put work in front of everything. Our culture here, unfortunately, is work. Not for all; for those who have been here for several years and have a good and stable life, it is different”.

This participant acknowledged her life and the life of Brazilians she knows is all about work, because she refers to “we Brazilians.” However, there is a hope for stability that comes with time in the U.S. Thus, time holds the key for enjoying the benefits of immigration for those who do not return to Brazil.

These harried routines and 1st generation immigrants’ attitudes towards their work experiences do not go unnoticed by 1.5 generation Brazilians. In fact, their description of what they see in their homes and community mirror older Brazilians’ statements.

“I notice older generations of Brazilians people always complain that in America, you become American because you go to work, come home, eat, sit in the couch, watch T.V., go to sleep, and repeat. And in Brazil you don’t do that, people go out, people move, there is always something to do. So, you become Americanized when you live here because their culture is more overwhelming than our culture”.

Dominican immigrants’ experiences with the rhythm of work in the U.S. were similar to the Brazilian immigrants’ experiences. An elder Dominican participant remarked:

“People begin to have a routine that is not normal for human beings. To be human is not to work from 9 to 5 and 9 to 5, go to home and sleep and return to work. This routine is for a robot”.

Most participants across CCs experienced work as the main activity in their lives. A low-income, Dominican participant expressed this well when she said that “Here other than your kids there is just work.” Dominicans seemed to be more concerned with dedicated time for family and collective activities. Many quotes reflect the impact of work and migration on family functions. For example, a participant in the barbers’ Conversation compared day to day lives here and there:

“You, if you are an immigrant here you have to work more, here you see your children less. There you have an economy where people have one job and work from 8 in the morning until 5 in the afternoon. There [DR] you arrive in your house and can-do family activities, on the weekends there are family activities and there is not this rhythm of full time, part time here it is work fulltime then part time and you don’t see your family”.

The lack of time for leisure seems to be beyond time itself. The work pressures were perceived by Dominican immigrants as an American cultural trait. A Dominican barber noted, “…clearly there is a difference [in culture] when you are in this country, there [DR] you enjoy life, here you work.” Brazilians and Dominicans both agreed that the American work culture is more accelerated than in their native countries, impacting several aspects of their lives. Brazilians concentrated on the grueling routine while Dominicans connected American work culture to impacts on family life.

### 3.3. American Dream

Both communities talked about the struggle of immigration.

“When you immigrate you have to sacrifice yourself to a cleaning job or whatever you can find, then you can’t go to school [because of the number of hours you have to work to get by]. You have to leave all of your dreams where you leave them…”

Dominican—Elder

“I wanted to come here and enlist in the army [28 years ago]. But when I arrived in Connecticut (…) they said I entered the country illegally so I had to return to enlist. (…) You come here with a dream and think it will work. It did not work. But what difference does it make? I continue working the same way landscaping, restaurants, cleaning jobs in supermarkets, house cleaning. I am here to this day”.

Brazilian—Construction Worker

Both of these participants expressed unrealized dreams. The younger generations in both CCs recognized the sacrifices of their parents. The 1.5 generation participants, because of this, felt obligated to succeed in America. One Brazilian participant offered:

“I felt like, often in my Brazilian group of Brazilian friends I was the only one dreaming big. Like all these Brazilian kids were like, yeah, my dad works as a carpenter, my dad works as a painter, I am just going to be a painter, carpenter or work in construction, live paycheck to paycheck. And I was like, well you are in America. Your parents brought you here for an opportunity. Are you going to waste that? Why are you limiting yourself to this?”

Brazilian—1.5 Generation

The notion that there is an “opportunity” to be taken advantage off and that it is up to the individual to make that effort was present in the narratives of participants across several CCs.

“Everyone has equal opportunities and you have respect for all groups and respect for all different ways of living that you can put into practice. The notion of entrepreneurship, that is, that you are able to succeed through your own effort, do your own work. These characteristics I think you can exercise perfectly. I think it’s a desirable thing to happen in Brazil too”.

Brazilian—professional

The American dream was often seen as a silver lining in the discussions of life in the U.S. for Brazilians. Participants discussed in many CCs their possibilities and hopes for a better future in the U.S. The following Brazilian 1.5 generation participant describes how despite not being ‘given’ material things that their peers had, they had to work to earn those things.

“I think growing up and having Brazilian and American culture, you learn really quickly that you are different. Even though you can assimilate to some other group, you know that you are not the same. Growing up in a wealthy neighborhood and knowing my mom was a single mother and trying to start a business, working a lot. Me and my brother we had a lot of free time so we would go to school and be like these kids have this, these kids have money, so at a young age we started working, 12 or 13 and we would work all summer. So, next think you know, we had everything they had. We had the shoes, we had the clothes, we had anything we wanted because we worked hard for it. We didn’t just sit there and be like, I am an immigrant, I just can’t. No, we would get what we wanted because we would work for it”.

Brazilian—1.5 generation

According to this participant, hard work distinguishes the haves and have nots. Brazilian participants were hopeful that hard work in America would pay off, and for those in the 1.5 generation conversations, that the sacrifices of their parents could not have been in vain.

Dominican participants were very confident about their capacity to contribute to the American work landscape.

“We, Dominicans have the capacity to do the job that is there, if we have to wash we will wash, if we have to iron, we will iron, if we have to fly a plane we will fly a plane”.

Dominican—Elder

However, in contrast with Brazilian immigrants, Dominicans were more aware of systemic inequalities that disadvantage immigrants and the fragile conditions that many families live in while in the U.S.

“All the systems are set up to make you fail. If you don’t have at least a middle-class income so that’s why you get, its easy no offense, I just lost my job in June, what was the first thing I thought well I get laid off, okay I got to give up my apartment I got to put my student loans on hold (…). In the Dominican Republic, you lose your job you’re like okay, well I’m going to sell mangoes on the street and oranges that I grow on my tree. So, I think that’s why it is easy to catch depression here because the moment you lose something that you control or that keeps you steady you freak out because it’s hard to get back on your feet”.

Dominican—1.5 Generation

Dominican and Brazilian immigrants seem to understand the struggles of immigrant work life similarly. While Brazilians seem to hold on to the promise of progress for hard work, Dominican immigrants may have been in the country for a longer period of time, and concluded that the work environment in the U.S was not designed for the benefit of hard-working immigrants.

### 3.4. Working Conditions

Precarious working conditions were a direct topic of discussion for the Brazilian Domestic Worker CC and the Construction Workers CC groups and a less frequent, but still present, theme for the other CCs.

“I work with plaster (…) you fill half tank, half a gallon of water, you take the bag that is the pure powder, you throw the gallon, the powder comes on your face. (…) I do not wear a mask, so like that, I see this too often”.

Brazilian—Construction Worker

Domestic workers had similar problems dealing with chemical cleaning products daily and not being provided with adequate protection gear to do their work safely.

“One day, we went to wash a bathroom, she said, ‘this here is the disposable glove, because I like to wear a disposable, not the yellow glove, okay’. She gave me one glove. I looked at her, I said, ‘Well, how am I going to use only one glove? I need both hands protected’.”

Brazilian—Domestic Worker

For some Brazilian participants, workplace hazards were compounded by their legal status. In fact, legal status was a salient theme in all CCs with Brazilians, as it had a great impact in several areas of immigrant life. When it comes to work, legal status is a risk factor for exploitation, leaving Brazilian immigrants vulnerable to practices such as wage inequities.

“Even being an immigrant, I could tell that some of the jobs I got at first was because of my status and they kind of knew that, even my pay, I would stay there for so long, and I just finally get to a point that I am like, you hit the glass roof. They don’t want to pass that point and you look over and you see Americans kids that are just starting to work and you have been there for five years and the same pay rate”.

Brazilian—1.5 Generation

Brazilians lack of documentation is a risk factor for workplace exploitation. The Brazilian CC elicited stories of denials of credit cards because of legal status, not accessing healthcare because of legal status, and being pulled over by the police and left in the street with a child while his car was towed due to lack of a driver’s license. The Brazilian 1.5 generation could see their parents struggle having to work and driving without a license. They actually inherited the same issues because of their undocumented status.

“My mom was always working and we needed to get around, so next thing you know you are driving. And then, you are the only 16-year-old out there that knows how to explain to a cop why you are driving without a license”.

Brazilian—1.5 Generation

Dominican CCs that discussed work conditions were more likely to talk about discrimination in the workplace. For example, a Dominican elder noted that she felt discriminated against and was denied work opportunities when she first arrived in the country because of her poor English proficiency.

“It happened to me the first time I came to this country, I went to work in an American company speaking very little English. (…) The American tells me one day, ‘I’m sorry you do not work more because you do not speak English’ and I have a knife in my hand to split a biscuit on an aluminum table, and I give him the knife and I told him ‘Fuck you!’ You speak English, if you give me a chance, [I can learn]. And he says ‘What did she say? What did she say?’ asks the man and said ‘I do not know what she said.’ ‘Yeah, you know what she said’ and I said ‘You know what I said, fuck you’ and I dropped my knife and left”.

Dominican—Elder

In addition, Dominican immigrants spoke more than Brazilians about the health impacts of the work conditions. Across CC groups, Dominican participants described the taxing situations that immigrants face as cultural and environmental changes post-migration impose challenges to physical and mental health.

### 3.5. Work and Health

Mental health was a shared concern for Dominican participants in the CCs. A Dominican professional said,

“…and the work, you know the stress from working…stress is normal but when you are doing more and more the stress becomes too much and then there is depression, you know.”

Depression, stress, anxiety were some common words in the narratives associated to being overworked, overtired and overwhelmed with responsibility without a safety net.

“(…) everything causes anxiety, you can’t meet your rent what are you going to do oh my gosh you’re sick today do I have childcare, now I am pregnant who’s going to take care of my kids I have to go to work, if I don’t pay my light bill I can’t turn on my lights so everything is just money that you have to pay out and if you don’t have money to pay any of those things then what I am trying to get at is everything causes anxiety then you get so depressed about everything”.

Dominican—Generation 1.5

While Dominicans were very aware of mental health, Brazilians discussed mostly the impact of the fast-paced work rhythm on their diets and physical health. One participant told us a story of a friend who was not able to eat well while at work, which had a profound effect on her health.

“She ate really badly because she couldn’t [eat] in the “go, go” of work, she ate very little. Very little. She had a problem of ulcerative colitis (…) she was hospitalized, she had several surgeries, I was in the hospital with her. And she ate very badly. Now she cannot eat right because there are things she cannot eat”.

Brazilian—Domestic Worker

Brazilians immigrants also talked about the adaptation to American foods as a threat to their health in the workplace.

“I go to work sometimes and eat a pizza, but I’m not going to stick with it, but I get home when I’m not working, my wife is home, my daughter, it’s rice, beans and meat. (...) When I eat a sandwich that I stay the whole day I do not feel [well]...I feel weak that you can already see that my health is not good, but now when I eat my food from the Brazilian culture that is my rice, the beans, the meat I feel [good]”.

Brazilian—Construction Worker

A Brazilian professional mentioned mental health in the context of legal status.

“I have a friend of mine she has panic attacks. She went to the doctor for her panic attacks because she is housecleaning and she cannot drive, she cannot...because afraid of the police”.

Brazilian—Professional

Dominicans also mentioned food and the impact of working hours on being less able to share meals with family. While their perspectives on the health impacts of work were quite similar, Brazilian and Dominican differences in priorities and perceptions may be due to differences in the length of stay in the U.S. and other contextual and cultural reasons.

## 4. Discussion

Brazilians’ and Dominicans’ culturally shaped expectations of what makes a good life contrasted considerably with the particulars of American work culture. This dissonance was a source of distress and disappointment for several participants. Our results are mostly aligned with previous research with Brazilian immigrants revealing that Brazilians experience a work–life imbalance in the U.S. as compared to their birth country, in addition to some adverse health effects related to their working conditions [26,41]. Brazilian and Dominican immigrants, particularly the working-class participants, are immersed in similar social environments and, according to their narratives, are exposed to similar working condition: long hours, low paying jobs and hazardous exposures were risk factors identified by both populations on our CC groups. These findings are supported by previous research that explored occupational health risks for Brazilian immigrants [4,17].

Although they work in similar environments, both populations have different expectations for life in the U.S. and understandings of how the structural realities affect their opportunities. Brazilians’ narratives often reveal hope for the future amidst the struggle and the belief in the promise of the American dream, while Dominicans have a soberer view of their current situation and a pessimistic forecast about the future. Brazilian 1.5 generation participants carried a heavy burden of what they perceived as their parents’ sacrifices, so that they could have a better life, which might have implications for mental health [49]. Dominicans in our CCs showed less trust in progress highlighting the difficulties and structural inequities in the U.S.

Dominicans in our sample, in accordance with previous research [50], were more likely to cite episodes of perceived workplace discrimination than Brazilians. Previous research has shown that perceptions of discrimination in Latino immigrant populations increase with education, assimilation and time in the U.S. [51]. Our findings could be partially explained by the fact that Dominicans have a longer tenure in the U.S. than Brazilian immigrants and have likely had longer exposure to episodes of discrimination. Another reason for different perceptions of discrimination could be differences in the racialization of Brazilians and Dominicans in the U.S.

Many Brazilian immigrants’ adaptation strategy is to reject a Latino identity because of the stigmas tied to undocumented status, and the conflation of undocumented status and Latino identity. As such, many Brazilians try to “become American” by passing as white or black [52]. Brazilian immigrants’ narratives included several mentions of documentation status as a barrier to work progress and life. However, the strategy and possibility of “hiding in plain sight” by becoming American may serve as a protective factor for the population. Most Dominicans identify themselves in variations of the identity “Indio,” which means Indian, in conjunction to a gradient of skin color i.e., “claro” (light), “escuro” (dark), “quemado” (burned) in the DR context and Latino in the American context [53]. However, many Dominicans are racialized as Black, even though many Dominicans do not identify in this way. This has implications for their experiences of discrimination [44,53]. Further research is needed to better understand how the processes of assimilation and integration, as they intersect with racialization, influence Brazilian and Dominican experiences of discrimination in the workplace.

Dominican immigrants in our CCs discussed mental health impacts of the structural inequities in the U.S. work environments. Dominican participants in our CCs discussed stress, anxiety and depression as a consequence of the changes in work routines and the impact of work on time with family and friends. They also described workplace discrimination as a common issue, which may also have implications for stress. Previous research has documented the association between mental health and discrimination in the Dominican populations [50,54]. Brazilian immigrants in our groups were more concerned with physical health through changes in diet and eating behaviors, and work and chemical hazard exposure. Yet, previous studies have found that between 35.3% and 51.2% of Brazilian immigrant respondents presented symptoms of depression [55,56]. Also, research using qualitative interviews with Brazilian migrants returned to Brazil retrospectively described their immigrant lives in the U.S. revealed high levels of stress and anxiety while living without legal documentation in the U.S. [36]. Mental health was not a strong theme for Brazilian immigrants on our CCs. However, work was not the primary focus of this research, it may be that further analysis reveals concerns related to mental health among Brazilians as well as Dominicans or, perhaps, the longer tenure of Dominicans have in the U.S. has implications for the openness with which our sample discussed mental health. Similarly, Brazilians’ positive views of immigration and the ambition to achieve the American dream serve as a protector to this population. Future studies should test this hypothesis.

This study has several limitations. The original study was not designed specifically to explore the work experiences of Brazilian and Dominican immigrants. However, work was so central to the lives of the immigrants in our sample that narratives were rich in details and meaning. Nonetheless, more in-depth research is needed to fill the gaps that we could not explore. Furthermore, we collected limited demographic information about participants beyond their eligibility traits for the cultural conversations. Future studies should investigate within-group differences in work experiences among immigrant populations. Also, conversations with the 1.5 generation are not representative of all Brazilian and Dominican 1.5 generation immigrants since all participants in our sample were college-bound. The views of 1.5-generation immigrants are important because they inherent their parents’ immigration status and cultural background as well as the experience of living within the mainstream culture.

## 5. Conclusions

This paper describes the results of an exploratory study that used a novel approach to focus groups inspired by Paulo Freire’s dialectical critical pedagogy [47]. Cultural conversations were democratic, fluid and, despite the lack of direct prompts, discussions related to work and the relationship of work and health came out in most CCs. The placement of Brazilian and Dominican immigrants in the labor market, their exposures and their perceptions of wellbeing may have an impact on these populations’ health. The process by which the health of Latinos declines over time possibly operates through such structural factors as work routines that limit access to adequate nutrition, social support and leisure. Further research should investigate the relationship between work and the Latino Epidemiologic Paradox for Brazilians and Dominicans [57,58].

The present paper contributes to our qualitative understanding of how Brazilian and Dominican immigrants make sense of their experiences working in the U.S. Brazilians are overworked but hopeful while Dominicans are more likely to express disenchantment with the immigration experience and related discrimination. We were able to uncover some indications of how these experiences may have an impact on Brazilian and Dominican immigrants’ physical and mental health. This research will inform policy makers, practitioners and those interested in improving the work–life balance for Brazilian and Dominican immigrants as well as future studies on culturally appropriate interventions aimed at improving overall immigrant health and wellbeing.

## Figures and Tables

**Table 1:** NVivo word query—text search.

NVivo Word Query—Text Search	
Brazilian CC transcripts (*n* = 5)	work, working, job, employer, co-worker, trabalho, money, emprego, trabalhando, dinheiro, busy, bisado, bizy, doente, sick
Dominicans CC transcripts (*n* = 5)	work, working, job, employer, co-worker, trabajo, trabajando, trabajar, money, dinero, empleado, enfermo, sick

**Table 2:** Brazilian and Dominican cultural conversations.

Brazilian		Dominicans	
Characteristics	*n*	Characteristics	*n*
Professionals	8	Professionals	6
1.5 Generation	3	1.5 Generation	4
Domestic Workers	7	Low wage workers	10
Construction Workers	8	Barbers	8
Elders (60+)	22	Elders	12
Total	48	Total	40

## References

[1] Lipscomb H.J., Loomis D., McDonald M.A., Argue R.A., Wing S. (2006). A conceptual model of work and health disparities in the United States. Int. J. Health Services.

[2] Krieger N. (2010). Workers are people too: Societal aspects of occupational health disparities—An ecosocial perspective. Am. J. Ind. Med..

[3] Siqueira C.E. (2012). Immigrant Worker Conference Report.

[4] Siqueira C.E., Jansen T. (2012). Working conditions of Brazilian immigrants in Massachusetts. J. Immigr. Minor. Health.

[5] Panikkar B., Woodin M.A., Brugge D., Desmarais A.M., Hyatt R., Gute D.M. (2013). Occupational health outcomes among self-identified immigrant workers living and working in Somerville, Massachusetts 2006–2009. J. Immigr. Minor. Health.

[6] Roelofs C., Sprague-Martinez L., Brunette M., Azaroff L. (2011). A qualitative investigation of Hispanic construction worker perspectives on factors impacting worksite safety and risk. Environ. Health.

[7] Minkler M., Salvatore A.L., Chang C., Gaydos M., Liu S.S., Lee P.T., Krause N. (2014). Wage theft as a neglected public health problem: An overview and case study from San Francisco’s Chinatown district. Am. J. Public Health.

[8] Casper W.J., Harris C., Taylor-Bianco A., Wayne J.H. (2011). Work–family conflict, perceived supervisor support and organizational commitment among Brazilian professionals. J. Voc. Behav..

[9] Salas-Wright C.P., Robles E.H., Vaughn M.G., Córdova D., Pérez-Figueroa R.E. (2015). Toward a typology of acculturative stress: Results among Hispanic immigrants in the United States. Hisp. J. Behav. Sci..

[10] Marcelli E.A., Holmes L., Estella D., Da Rocha F., Granberry P., Buxton O. (2009). (In)Visible (im)Migrants: The Health and Socioeconomic Integration of Brazilians in Metropolitan Boston.

[11] U.S. Bureau of Labor Statistics Economic News Release: Table 6. Employed persons working at home, workplace, and time spent working at each location by full- and part-time status and sex, jobholding status, and educational attainment, 2017 annual averages. https://www.bls.gov/news.release/atus.t06.htm.

[12] Landsbergis P.A., Choi B., Dobson M., Sembajwe G., Slatin C., Delp L., Baron S. (2018). The key role of work in population health inequities. Am. J. Public Health.

[13] Heaven B.E.N., Brown L.J., White M., Errington L., Mathers J.C., Moffatt S. (2013). Supporting well-being in retirement through meaningful social roles: Systematic review of intervention studies. Milbank Q..

[14] Matz-Costa C., Besen E., Boone James J., Pitt-Catsouphes M. (2012). Differential impact of multiple levels of productive activity engagement on psychological well-being in middle and later life. Gerontologist.

[15] CDCP-NIOSH Stress… at Work. https://www.cdc.gov/niosh/docs/99-101/default.html.

[16] Marmot M., Theorell T. (1988). Social class and cardiovascular disease: The contribution of work. Int. J. Health Serv..

[17] Siqueira C.E., Roche A.G. (2013). Occupational health profile of Brazilian immigrant housecleaners in Massachusetts. J. Environ. Occup. Health Policy.

[18] Ahonen E.Q., López-Jacob M.J., Vázquez M.L., Porthé V., Gil-González D., García A.M., Benavides F.G. (2010). Invisible work, unseen hazards: The health of women immigrant household service workers in Spain. Am. J. Ind. Med..

[19] Malhotra R., Arambepola C., Tarun S., de Silva V., Kishore J., Østbye T. (2013). Health issues of female foreign domestic workers: A systematic review of the scientific and gray literature. Int. J. Occup. Environ. Health.

[20] Tsai J.H.C., Salazar M.K. (2007). Occupational hazards and risks faced by Chinese immigrant restaurant workers. Family Commun. Health.

[21] Quach T., Gunier R., Tran A., Von Behren J., Doan-Billings P.A., Nguyen K.D., Reynolds P. (2011). Characterizing workplace exposures in Vietnamese women working in California nail salons. Am. J. Public Health.

[22] Hansen E., Donohoe M. (2003). Health issues of migrant and seasonal farmworkers. J. Health Care Poor Underserved.

[23] Flores L.Y., Mendoza M.M., Ojeda L., He Y., Meza R.R., Medina V., Jordan S. (2011). A qualitative inquiry of Latino immigrants’ work experiences in the Midwest. J. Couns. Psychol..

[24] Eggerth D.E., DeLaney S.C., Flynn M.A., Jacobson C.J. (2011). Work experiences of Latina immigrants: A qualitative study. J. Career Dev..

[25] Panikkar B., Brugge D., Gute D.M., Hyatt R.R. (2015). “They See Us As Machines:” The Experience of Recent Immigrant Women in the Low Wage Informal Labor Sector. PLoS ONE.

[26] Messias D.K.H. (2001). Transnational perspectives on women’s domestic work: Experiences of Brazilian immigrants in the United States. Women Health.

[27] Viruell-Fuentes E.A., Miranda P.Y., Abdulrahim S. (2012). More than culture: Structural racism, intersectionality theory, and immigrant health. Soc. Sci. Med..

[28] U.S. Census Bureau Selected Population Profile in the United States. American Community Survey 1-Year Estimates. Foreign-born Brazilians in Massachusetts. https://factfinder.census.gov/faces/tableservices/jsf/pages/productview.xhtml?pid=ACS_17_1YR_S0201&prodType=table.

[29] U.S. Census Bureau Selected Population Profile in the United States. American Community Survey 1-Year Estimates. Foreign-born Dominicans in Massachusetts. https://factfinder.census.gov/faces/tableservices/jsf/pages/productview.xhtml?pid=ACS_17_1YR_S0201&prodType=table.

[30] Levitt P. (2001). The Transnational Villagers.

[31] Margolis M.L. (1995). Transnationalism and popular culture: The case of Brazilian immigrants in the United States. J. Pop. Cult..

[32] Lima A., Siqueira C.E. (2007). Brazilians in the us and Massachusetts: A Demographic and Economic Profile.

[33] Blizzard J.B., Batalova J. Brazilian Immigrants in the United States. https://www.migrationpolicy.org/article/brazilian-immigrants-united-states.

[34] U.S. Census Bureau Selected Population Profile in the United States. American Community Survey 1-Year Estimates. Foreign-born Brazilians in the U.S.. https://factfinder.census.gov/faces/tableservices/jsf/pages/productview.xhtml?pid=ACS_17_1YR_S0201&prodType=table.

[35] Passel J.S., D’Vera C. (2016). Overall Number of US Unauthorized Immigrants Holds Steady since 2009.

[36] Joseph T.D. (2011). “My life was filled with constant anxiety”: Anti-immigrant discrimination, undocumented status, and their mental health implications for Brazilian immigrants. Race Soc. Probl..

[37] Gute D.M., Siqueira E., Goldberg J.S., Galvão H., Chianelli M., Pirie A. (2009). The Vida Verde Women’s Co-Op: Brazilian immigrants organizing to promote environmental and social justice. Am. J. Public Health.

[38] Zong J., Batalova J. Dominican Immigrants in the United States. Migration Policy Institute. https://www.migrationpolicy.org/article/dominican-immigrants-united-states#IncomePoverty.

[39] U.S. Census Bureau Selected Population Profile in the United States. American Community Survey 1-Year Estimates. Foreign-born Dominicans in the U.S.. https://factfinder.census.gov/faces/tableservices/jsf/pages/productview.xhtml?pid=ACS_17_1YR_S0201&prodType=table.

[40] Reinman S.L. The World Fact Book: Central America Dominican Republic. https://www.cia.gov/library/publications/the-world-factbook/geos/dr.html.

[41] López G. (2013). Hispanics of Mexican Origin in the United States. http://www.pewhispanic.org/2015/09/15/hispanics-of-mexican-origin-in-the-united-states-2013.

[42] Marcelli E., Holmes L., Troncoso M., Granberry P., Buxton O., Hernández R. (2009). Permanently Temporary. The Health and Socioeconomic Status of Dominicans in Metropolitan Boston.

[43] Candelario G.E. (2000). Hair race-ing: Dominican beauty culture and identity production. Meridians.

[44] Araujo-Dawson B. (2015). Understanding the complexities of skin color, perceptions of race, and discrimination among Cubans, Dominicans, and Puerto Ricans. Hisp. J. Behav. Sci..

[45] Lee D.L., Ahn S. (2012). Discrimination against Latina/os: A meta-analysis of individual-level resources and outcomes. Couns. Psychol..

[46] Pessar P.R. (1984). The linkage between the Household and Workplace of Dominican Women in the US. Int. Migr. Rev..

[47] Freire P. (2018). Pedagogy of the Oppressed.

[48] Braun V., Clarke V. (2006). Using thematic analysis in psychology. Qual. Res. Psychol..

[49] Gonzales R.G., Suárez-Orozco C., Dedios-Sanguineti M.C. (2013). No place to belong: Contextualizing concepts of mental health among undocumented immigrant youth in the United States. Am. Behav. Sci..

[50] Araújo Dawson B., Panchanadeswaran S. (2010). Discrimination and acculturative stress among first-generation Dominicans. Hisp. J. Behav. Sci..

[51] Pérez D.J., Fortuna L., Alegria M. (2008). Prevalence and correlates of everyday discrimination among US Latinos. J. Commun. Psychol..

[52] Marrow H. (2003). To Be or Not to Be (Hispanic or Latino). Ethnicities.

[53] Itzigsohn J., Giorguli S., Vazquez O. (2005). Immigrant incorporation and racial identity: Racial self-identification among Dominican immigrants. Ethnic Racial Stud..

[54] Araújo B.Y., Borrell L.N. (2006). Understanding the link between discrimination, mental health outcomes, and life chances among Latinos. Hisp. J. Behav. Sci..

[55] Lazar-Neto F., Louzada A.C.S., de Moura R.F., Calixto F.M., Castro M.C. (2017). Depression and Its Correlates Among Brazilian Immigrants in Massachusetts, USA. J. Immigr. Minor. Health.

[56] Sánchez M., Cardemil E., Adams S.T., Calista J.L., Connell J., DePalo A., Melo T. (2014). Brave new world: Mental health experiences of Puerto Ricans, immigrant Latinos, and Brazilians in Massachusetts. Cult. Divers. Ethnic Minor. Psychol..

[57] Cho Y., Frisbie W.P., Hummer R.A., Rogers R.G. (2004). Nativity, duration of residence, and the health of Hispanic adults in the United States 1. Int. Migr. Rev..

[58] Stephen E.H., Foote K., Hendershot G.E., Schoenborn C.A. (1994). Health of the foreign-born population: United States, 1989–1990. Adv. Data.

